# Supplementary material for: A robust semi-supervised NMF model for single cell RNA-seq data
Source: PeerJ. 2020 Oct 16;8:e10091. doi: 10.7717/peerj.10091 (PMC7571410; doi:10.7717/peerj.10091)
Supplement: Supplemental Information 2 [file peerj-08-10091-s002.docx]

Supplementary Material for

“A robust semi-supervised NMF model for

single cell RNA-seq data”

## January 5, 2020

**Contents**

1. [**Enrichment analysis for feature genes 2**](#_TOC_250002)
2. **Definition of Adjusted Rand Index 3**
3. [**Supplementary Figures 3**](#_TOC_250001)
4. [**Supplementary Tables 6**](#_TOC_250000)

# Enrichment analysis for feature genes

The enrichment analysis method is based on hypergeometric distribution, which is often used to describe the probability distribution of sampling test. For ex- ample, in $N$ commodities, there are $M$ designated commodities, sampling $n$ commodities without replacement and the distribution of the designated com- modities been sampled, i.e. *X* ∼ *H*(*N, n, M* ), and the probability of $k$ desig- nated commodities been sampled is:

∼

$$p\left( k \right)=P\left( X=k \right)$$

$$=\frac{\binom{M}{k}*\binom{N-M}{n-k}}{\binom{N}{n}}$$

Applying this model to the gene set enrichment analysis, *N* is the total number of genes in KEGG database, *M* is the number of genes belonging to a specific pathway in the database, and *n* is the number of feature genes in the cell cluster that we need to analyze. $k$ is the number of genes belonging to a specific pathway in $n$, so we can calculate whether the gene set *n* is enriched in a certain pathway. However, this probability cannot be directly used as the result of enrichment analysis. It is necessary to consider the random situation and perform a statistical significance test, such as hypothesis test, and calculate the p-value. The p-value or significance is, for a given statistical model, the probability that, when the null hypothesis is true, the statistical summary (such as the absolute value of the sample mean difference between two compared groups) would be greater than or equal to the actual observed results. If the p-value is very small, the probability of this situation is very small, and if it occurs, according to the principle of small probability event, we have reason to reject the original hypothesis, the smaller the p-value, the more reason we reject the original hypothesis. In short, the smaller the p-value, the more significant the results are. But whether the test results are ”significant”, ”moderately significant” or ”highly significant” needs to be solved by ourselves according to the practical problems. In our enrichment analysis, the p-value is calculated from the following formula:

$$p-value=\sum_{i=m}^{M} \frac{\binom{M}{i}*\binom{N-M}{n-i}}{\binom{N}{n}}$$

$$=1-\sum_{i=0}^{m-1} \frac{\binom{M}{i}*\binom{N-M}{n-i}}{\binom{N}{n}}$$

We can understand that the p-value represents the sum of the probability that $m$ genes are observed in the pathway and more extreme than that, so $i$ is from $m$ to $M$ . Considering that for now there are 530 pathways in KEGG database, 530 hypothesis tests will cause a high degree of false positivity, so we also need multiple hypothesis test correction. Here we use false discovery rate (FDR) correction and calculate the q-value. A q-value is defined as the minimun FDR

that can be attained when calling that ”feature” significant. For instance, q- value of 5% means that 5% of significant results will result in false positive.

# Definition of Adjusted Rand Index

$$ARI=\frac{\sum_{ij} \binom{n_{ij}}{2}-[\sum_{i} \binom{a_{i}}{2}\sum_{j} \binom{b_{j}}{2}]/\binom{n}{2}}{\frac{1}{2}[\sum_{i} \binom{a_{i}}{2}+\sum_{j} \binom{b_{j}}{2}]-[\sum_{i} \binom{a_{i}}{2}\sum_{j} \binom{b_{j}}{2}]/\binom{n}{2}}$$

Where $\binom{}{}$ is a binomial coefficient, $n_{ij}$ are values from the contingency table, $a_{i}$ and $b_{j}$ denote the summation of the $i$th row $j$th column of the contingency table respectively.

# Supplementary Figures


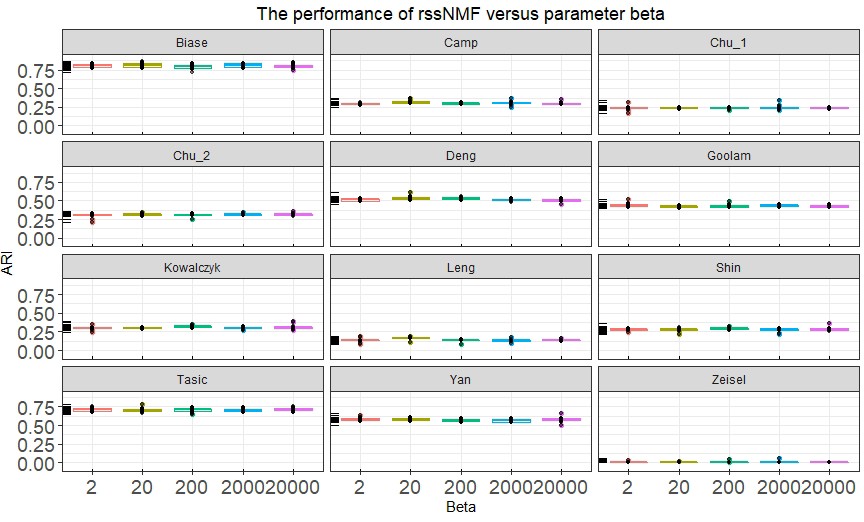


Supplementary Fig.1: Performance of rssNMF versus parameter *β*. The rss- NMF performs well when *β* varies with in a certain range, but the accuracy drops drastically when *β* is too large.


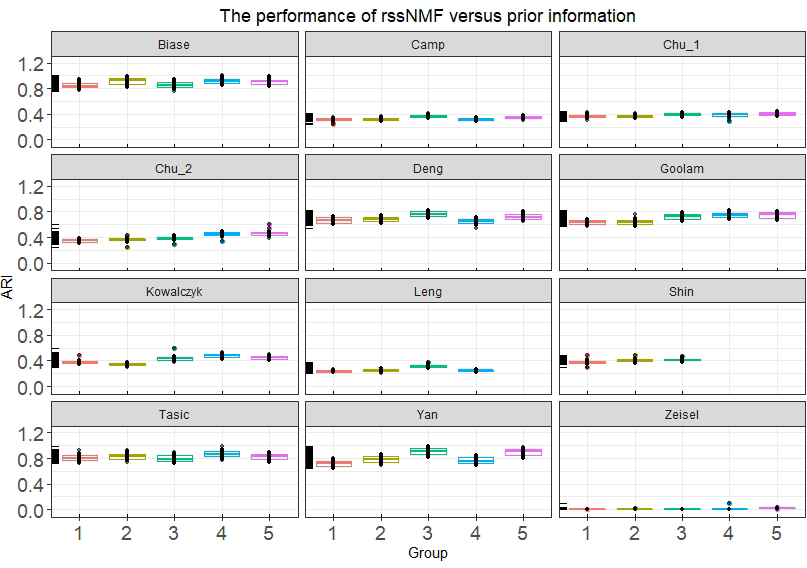


Supplementary Fig.2: Performance of rssNMF versus marker genes of different number of groups. We set the group number from 1 to 5 and the dataset Leng and Shin have only four and three clusters respectively at all.


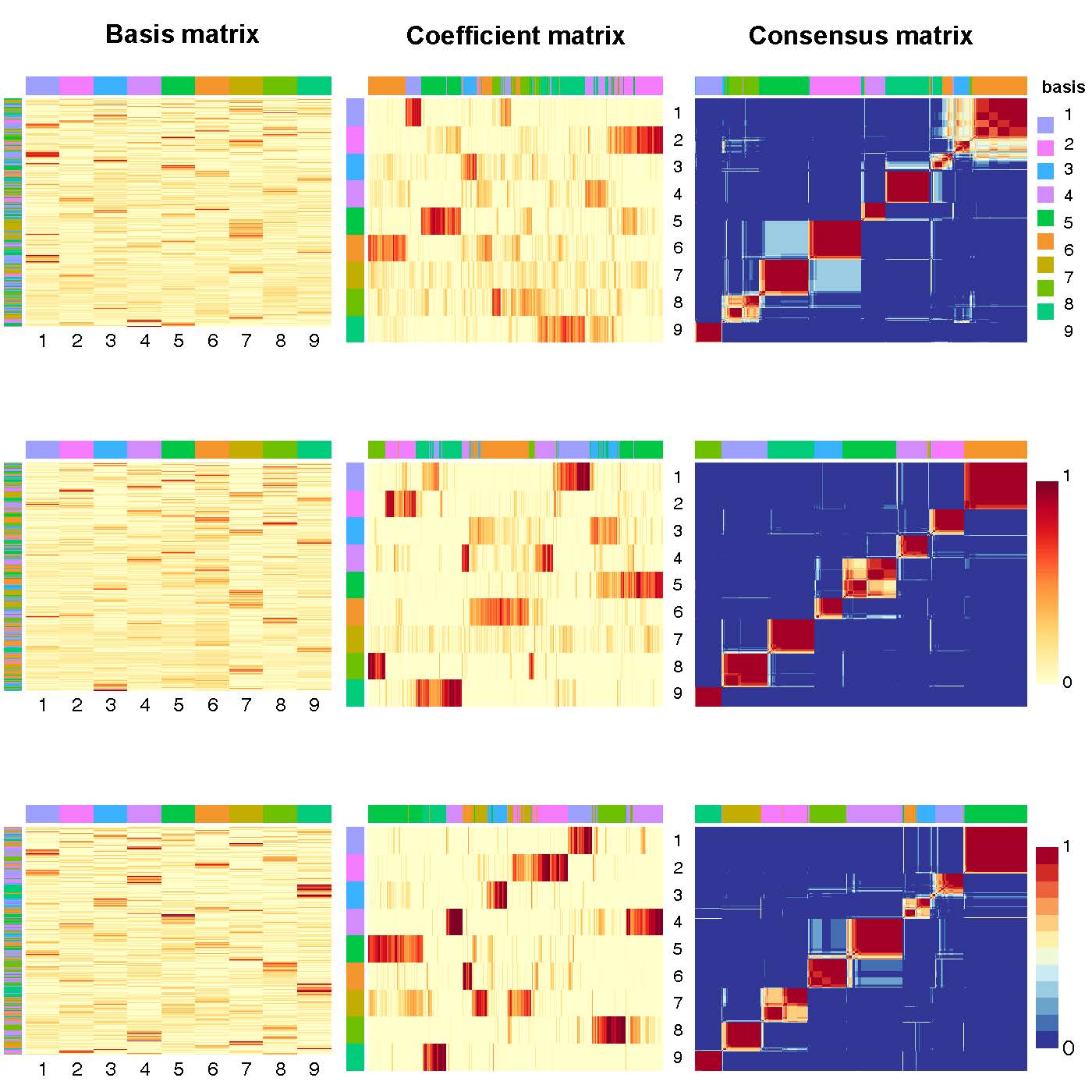


Supplementary Fig.3: $W$ (basis matrix, left), $H$(Factorizing matrices $W$ (basis matrix, left), $H$ (coefficient matrix, middle) and consensus matrix (right) respectively obtained from NMF (top row), rNMF (middle row) and rssNMF (bottom row) for dataset 8 with 734 cells and 9 clusters. The annotation color bar denotes 9 clusters. The rows annotation of $W$ and columns of $H$ indicate the assignment of genes and samples for clusters. The parameter $\alpha= 2$ for rNMF and rssNMF and $\beta= 2$ for rssNMF.


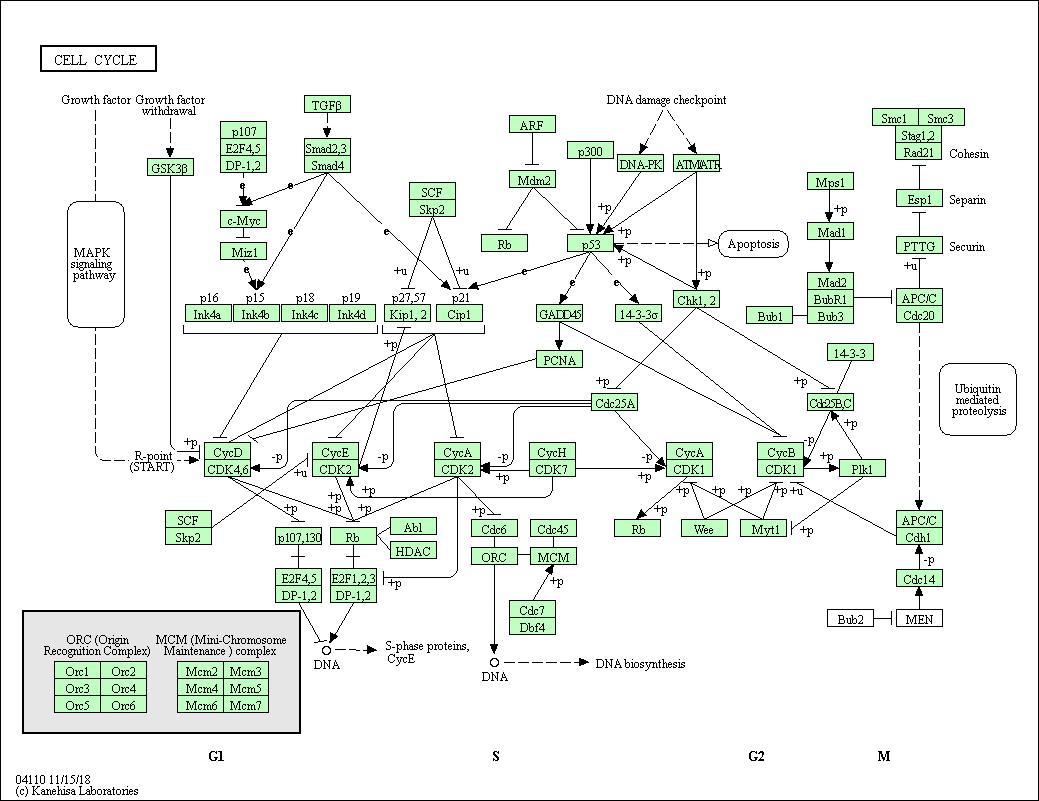


Supplementary Fig.4: A schema of pathway Hsa04110 in KEGG database. Hsa04110 is a pathway map of mitotic processes in human cell cycle, which consists of a series of interacting proteins. Green rectangular boxes denote specific proteins, white rounded rectangular boxes denote other pathways, white circles denote specific chemical molecules, and solid and dashed arrows denote different types of protein-protein interactions.

# Supplementary Tables

All datasets into one class, which is embryonic cells of human or mouse. For embryonic cells, we can easily find the maker genes from various databases. Class includes dataset Yan, Leng, Camp, Chu 1 and Chu 2 which are human embryonic cells and dataset Biase, Goolam, Shin, Deng and Kowalczyk which are mouse embryonic cells.

The first is five human embryonic cell datasets. Yan was obtained by se- quencing human donated oocytes and embryos consisted of 90 individual cells from 20 oocytes and embryos (Yan *et al.*, 2013). The embryos were at seven cru- cial stages of preimplantation development: metaphase II oocyte, zygote, 2-cell, 4-cell, 8-cell, morula and late blastocyst at hatching stage. Leng is a single-cell RNA-sequencing dataset of human embroyonic stem cells (Leng *et al.*, 2015). Total 213 H1 single cells and 247 H1-Fucci single cells were sequenced. The 213 H1 cells were used to evaluate Oscope in identifying oscillatory genes. The H1- Fucci cells were used to confirm the cell cycle gene cluster identified by Oscope in the H1 hESCs. Camp is a 734 single-cell transcriptomes from human fetal neocortex or human cerebral organoids from multiple time points (Camp *et al.*, 2015). Fetal neocortex data were generated from dissociated whole organoids derived from induced pluripotent stem cell line after the start of embryoid body culture. Chu 1 and Chu 2 come from the same study and are all expression profile of human pluripotent stem cells, which offer a unique cellular model to study lineage specifications of the primary germ layers during human development (Chu *et al.*, 2016). Chu 1 is 758 single cells from time course profiling and Chu 2 is 1018 single cells from snapshot progenitors.

For mouse embryonic cells in class one, Biase is the expression profile from mouse embryonic cells (Biase *et al.*, 2014). 9 zygotes, 10 2-cell, and 5 4-cell mouse embryos were collected and multi-cell embryos were separated into blas- tomeres. 4 inner cell mass and 3 trophectoderm samples are also extracted from mouse blastocysts. Goolam is from pre-implantation development of mouse em- bryos (Goolam *et al.*, 2016). Transcriptomes were determined for all blastomeres of 28 embryos isolated at the 2-cell, 4-cell, and 8-cell stages, and for individual cells at the 16-cell and 32-cell stages, corresponding to the morula and blasto- cyst, respectively. Deng is also from mouse preimplantation embryos and can be utilized to investigate allele-specific gene expression of embryos at single-cell resolution (Deng *et al.*, 2014). Shin is single-cell transcriptomes of adult hip- pocampal quiescent neural stem cells and their immediate progeny (Shin *et al.*, 2015). Kowalczyk has 564 mouse hematopoietic cells and was designed to exam- ine variation between individual hematopoietic stem and progenitor cells from two mouse strains as they age (Kowalczyk *et al.*, 2015).

Supplementary Tab. 1: Gene set enrichment analysis for feature genes of each cluster in dataset Yan

| Clusters Description | GeneRatio | q-value |
| --- | --- | --- |
| Proteasome | 9/148 | 2.41E-05 |
| DNA replication | 8/148 | 2.62E-05 |
| Mismatch repair | 5/148 | 0.002908 |
| Cluster 1 Phagosome | 11/148 | 0.005374 |
| Gap junction | 8/148 | 0.008104 |
| Aminoacyl-tRNA biosynthesis | 6/148 | 0.042427 |
| Glycolysis / Gluconeogenesis | 6/148 | 0.043363 |
| RNA transport | 12/85 | 2.09E-05 |
| Ribosome | 11/85 | 4.00E-05 |
| Spliceosome | 30/204 | 4.66E-18 |
| RNA transport | 28/204 | 1.06E-13 |
| Ribosome biogenesis in eukaryotes | 18/204 | 9.97E-09 |
| Cluster 4 mRNA surveillance pathway | 15/204 | 4.71E-07 |
| Cell cycle | 12/204 | 0.003329 |
| Oocyte meiosis | 11/204 | 0.010835 |
| Basal transcription factors | 6/204 | 0.021661 |
| Cluster 5 Ribosome | 21/32 | 1.15E-27 |
| Oxidative phosphorylation | 18/136 | 7.99E-10 |
| Thermogenesis | 18/136 | 2.78E-06 |
| Ribosome | 12/136 | 0.00029 |
| Terpenoid backbone biosynthesis | 5/136 | 0.000601 |
| Proteasome | 6/136 | 0.001978 |
| Cluster 6 Citrate cycle (TCA cycle) | 5/136 | 0.002263 |
| Cholesterol metabolism | 6/136 | 0.002876 |
| Protein processing in endoplasmic reticulum | 10/136 | 0.006297 |
| Endocytosis | 11/136 | 0.030963 |
| Carbon metabolism | 7/136 | 0.034118 |
| Pyruvate metabolism | 4/136 | 0.036368 |
| Ribosome | 27/74 | 1.23E-26 |
| Oxidative phosphorylation | 24/74 | 5.67E-24 |
| Thermogenesis | 25/74 | 1.67E-19 |
| Cardiac muscle contraction | 9/74 | 1.70E-07 |
| Retrograde endocannabinoid signaling | 11/74 | 4.34E-07 |
| Spliceosome | 8/74 | 0.00012 |
| Ribosome | 27/79 | 2.20E-25 |
| Cluster 8 Oxidative phosphorylation | 9/79 | 0.00013 |
| Thermogenesis | 9/79 | 0.006349 |
| Spliceosome | 21/206 | 6.55E-09 |
| Cell cycle | 14/206 | 0.000443 |
| Proteasome | 8/206 | 0.001338 |
| RNA degradation | 10/206 | 0.002024 |
| Oocyte meiosis | 12/206 | 0.003796 |
| RNA transport | 14/206 | 0.003796 |
| RNA polymerase | 5/206 | 0.035954 |
| Base excision repair | 5/206 | 0.042112 |

Supplementary Tab. 1 shows the results of enrichment analysis for feature genes of dataset Yan and provides the q-value of enrichment pathways. The enrichment analysis method we use is based on hypergeometric distribution. All pathways have a q-value less than 0.05 and we present some significant biological processes for each cluster.

# References

Biase, F., Cao, X., and Zhong, S. (2014). Cell fate inclination within 2-cell and 4-cell mouse embryos revealed by single-cell rna sequencing. *Genome research*, page gr. 177725.114.

Camp, J. G., Badsha, F., Florio, M., *et al.* (2015). Human cerebral organoids recapitulate gene expression programs of fetal neocortex development. *Pro- ceedings of the National Academy of Sciences*, **112**(51), 15672–15677.

Chu, L.-F., Leng, N., Zhang, J., *et al.* (2016). Single-cell rna-seq reveals novel regulators of human embryonic stem cell differentiation to definitive endo- derm. *Genome biology* , **17**(1), 173.

Deng, Q., Ramskold, D., Reinius, B., and Sandberg, R. (2014). Single-cell rna- seq reveals dynamic, random monoallelic gene expression in mammalian cells. *Science*, **343**(6167), 193–6.

Goolam, M., Scialdone, A., Graham, S. J., *et al.* (2016). Heterogeneity in oct4 and sox2 targets biases cell fate in 4-cell mouse embryos. *Cell* , **165**(1), 61–74.

Kowalczyk, M. S., Tirosh, I., Heckl, D., *et al.* (2015). Single-cell rna-seq reveals changes in cell cycle and differentiation programs upon aging of hematopoietic stem cells. *Genome research*, **25**(12), 1860–1872.

Leng, N., Chu, L.-F., Barry, C., *et al.* (2015). Oscope identifies oscillatory genes in unsynchronized single-cell rna-seq experiments. *Nature methods*, **12**(10), 947.

Shin, J., Berg, D. A., Zhu, Y., *et al.* (2015). Single-cell rna-seq with waterfall reveals molecular cascades underlying adult neurogenesis. *Cell stem cell* , **17**(3), 360–372.

Tasic, B., Menon, V., Nguyen, T. N., *et al.* (2016). Adult mouse cortical cell taxonomy revealed by single cell transcriptomics. *Nature neuroscience*, **19**(2), 335.

Yan, L., Yang, M., Guo, H., *et al.* (2013). Single-cell rna-seq profiling of hu- man preimplantation embryos and embryonic stem cells. *Nature structural & molecular biology* , **20**(9), 1131.

Zeisel, A., Mun˜oz-Manchado, A. B., Codeluppi, S., *et al.* (2015). Cell types in the mouse cortex and hippocampus revealed by single-cell rna-seq. *Science*, **347**(6226), 1138–1142.
